# Clinical indications of premenstrual disorders and subsequent risk of injury: a population-based cohort study in Sweden

**DOI:** 10.1186/s12916-021-01989-4

**Published:** 2021-05-26

**Authors:** Qian Yang, Arvid Sjölander, Yuchen Li, Alexander Viktorin, Elizabeth R. Bertone-Johnson, Weimin Ye, Fang Fang, Unnur A. Valdimarsdóttir, Donghao Lu

**Affiliations:** 1 Department of Medical Epidemiology and Biostatistics, Karolinska Institutet, Nobels Väg 12A, 17177 Stockholm, Sweden; 2 Department of Biostatistics and Epidemiology, School of Public Health and Health Sciences, University of Massachusetts Amherst, Amherst, MA-01003 USA; 3 Department of Health Promotion and Policy, School of Public Health and Health Sciences, University of Massachusetts Amherst, Amherst, MA-01003 USA; 4 Institute of Environmental Medicine, Karolinska Institutet, SE-171 77 Stockholm, Sweden; 5 Center of Public Health Sciences, Faculty of Medicine, University of Iceland, IS-101 Reykjavík, Iceland; 6 Department of Epidemiology, Harvard T.H. Chan School of Public Health, Boston, MA-02115 USA

**Keywords:** Premenstrual disorders, Injury, Suicidal behavior, Accidents, Cohort study

## Abstract

**Background:**

Premenstrual disorders, including premenstrual syndrome and premenstrual dysphoric disorder, are suggested to be correlated with suicidal behavior and accidents in cross-sectional and retrospective studies. However, prospective data are still lacking.

**Methods:**

We performed a population-based cohort study including 1,472,379 Swedish women of reproductive age who were followed from 2001 to 2012. Within the cohort, we also performed a sibling analysis where we compared the rates of injury between full sisters. By linking to the Patient and the Prescribed Drug Registers, we identified 18,628 women with any clinical indications for premenstrual disorders in the cohort (population analysis) and 7674 women in the sibling analysis. Any injury, primarily suicidal behavior (completed suicide and suicide attempt) or accidents (e.g., fall and transportation accidents), was identified through the Patient and Causes of Death Registers as the primary outcome. We estimated hazard ratios (HRs) and 95% confidence intervals (CIs) of these outcomes among women with premenstrual disorders in both population and sibling analyses using multivariable Cox proportional hazards regression.

**Results:**

During a maximal follow-up of 12 years (mean 9.55 years), we identified 2390 women with premenstrual disorders with any injury; 216 through suicidal behavior and 2191 through accidents. Compared to women without premenstrual disorders, women with premenstrual disorders were at increased risk of any injury (HR 1.37, 95% CI 1.31–1.42), particularly suicidal behavior (HR 2.26, 95% CI 1.97–2.59) and accidents (HR 1.32, 95% CI 1.27–1.38). Such associations somewhat attenuated yet remained significant in the sibling analysis (HRs: 1.31 for any injury, 1.86 for suicidal behavior, and 1.29 for accidents). Additional adjustment for psychiatric comorbidities minimally altered the associations with any injury and accidents in both population and sibling analyses, whereas the association with suicidal behavior was considerably attenuated to non-significance in the sibling analysis. Such risks were particularly strong within 2 years after receiving the diagnosis of premenstrual disorders and were evident among women with premenstrual disorders with and without psychiatric comorbidities.

**Conclusions:**

Our findings suggest that women with a clinical indication of premenstrual disorders are at increased subsequent risk of injury, particularly accidents within the first 2 years after diagnosis.

**Supplementary Information:**

The online version contains supplementary material available at 10.1186/s12916-021-01989-4.

## Background

Premenstrual disorders (PMD), characterized by affective and physical symptoms that cyclically occur 7–10 days before menstruation, affect millions of women of reproductive age worldwide. PMD include premenstrual syndrome (PMS) and premenstrual dysphoric disorder (PMDD). The former is defined by the American College of Obstetricians and Gynecologists (ACOG) [1], and the latter is an official diagnosis in the Diagnostic and Statistical Manual of Mental Disorders (DSM), characterized by pronounced affective symptoms [2, 3]. Both PMS and PMDD are associated with social impairment, which is often more substantial among patients with PMDD [2]. The estimated prevalence is 20 to 40% for PMS and 2 to 6% for PMDD [2, 4–7]. The chronic and cyclic condition may have a lasting impact on women’s quality of life [8]. Indeed, the level of impairment attributable to PMD has been suggested to be not much lower than in major depression [9]. Despite clear evidence for decreased physical and mental well-being among women with PMD [10], little is known about the impact on major health outcomes, such as injury, in this population.

Injury is a major public health concern among women of reproductive age. Intentional and unintentional injuries together account for approximately 15% of deaths in women aged 15–49 years in global studies [11]. It is however unclear to what extent injury is associated with PMD. PMD have been hypothesized to increase risk of suicide behavior, given the severity of cyclic depressive and anxiety symptoms associated with these conditions. Indeed, the results from a systematic review including 10 retrospective studies suggest that PMD were associated with suicide attempts [12]. This association was independent of psychiatric comorbidities for PMDD but not for PMS [13]. Furthermore, it is plausible that women with PMD are more prone to accidents due to the fatigue, irritability, and lack of concentration during the days before menstruation. In a cross-sectional study, PMD was associated with accidents, particularly during the days preceding and during menstruation [14]. Another retrospective study among female soccer players indicates a positive correlation between premenstrual symptoms and accidents [15]. However, these studies were conducted in relatively small and distinct populations, and mostly rely on retrospective assessments of premenstrual symptoms and/or injury, which may be prone to recall bias. Moreover, PMD and suicidal behavior have been reported to cluster within families [16, 17]. Because socioeconomic disadvantage and genetic susceptibility may confound the reported associations [16, 18, 19], prospective data combined with sibling analyses are needed to estimate the association of PMD and risk of injury. We assessed these relations in a Swedish population-based cohort, comparing the risk of injury in women with clinical indications of PMD to risk in the general population and in their full sisters.

## Methods

### Study design

We conducted a registry-based cohort study of 1,543,653 women who were born in Sweden from 1960 to 1990 and resided in Sweden in 1990, according to the Swedish Population and Housing Census. Using the unique personal identification numbers assigned to every resident, we cross-linked all women to the nationwide Causes of Death Register and Migration Register. All women were followed from January 1, 2001, or their 15 birthday (as 96% Swedish women would have had menarche by age 15 [20]), whichever occurred last, until death, emigration, bilateral oophorectomy, or hysterectomy, their 52nd birthday (the average age of menopause in Sweden [21]), or December 31, 2012, whichever occurred first. We excluded 71,346 women due to bilateral oophorectomy or hysterectomy (*n*=2698), emigration (*n*=64,689), or death (*n*=3,959) before cohort entry, leaving 1,472,379 women in the cohort (population analysis). The Swedish Multi-Generation Register consists of information on 97% of mothers and 95% of fathers of individuals born after 1932 and alive in 1961 [22]. By linking to the register, we identified 638,910 women who were full sisters (i.e., sharing the same biological parents) for the sibling analysis.

### Ascertainment of premenstrual disorders

Clinical indications of PMD were identified using any primary or secondary clinical diagnosis of PMD from the Patient Register (625E in International Classification of Diseases (ICD) 9th revision (ICD-9) and N943 in ICD-10). The Patient Register has nationwide coverage on inpatient care from 1987 onward and includes information on more than 80% of specialist-based outpatient visits from 2001 onward with high validity (positive predicted value of 85–95% across diseases) [23]. All PMD diagnoses obtained from the Patient Register are considered provisional, as we could not confirm that all providers of PMD diagnoses ascertained from the Patient Register used two cycles of prospective daily symptom ratings for diagnosing [3], although it is required in the Swedish healthcare guidelines for PMDD in many regions [24]. The Patient Register does not cover diagnoses made in primary care, and 49.3% of mental health conditions are treated in primary care in Sweden [9]. Therefore, we also identified PMD by searching the Prescribed Drug Register for any PMD diagnosis or clear indication of PMD treatment in prescriptions of anti-depressants (the Anatomical Therapeutic Chemical (ATC) code: N06AB, N06AX, N06AA) and oral contraceptives (ATC code: G03A, G02B). We included only prescriptions explicitly made for PMD treatment, as indicated by PMD diagnosis/prescription specific to PMD. Diagnostic codes and keywords indicated in prescriptions are described in supplementary Table S1. The register collects information on drugs redeemed with a prescription from all pharmacies in Sweden from July 2005 onward, including drugs prescribed in primary care [25]. We defined the date of PMD diagnosis as either the date of clinical diagnosis or the date that a prescription for PMD medication was filled, whichever came first.

PMD were treated as a time-varying exposure. Women who had not received a diagnosis of PMD during follow-up contributed person-time to the reference group. Women with PMD contributed person-time to the reference group from the start of follow-up until the date of diagnosis and contributed to the PMD group thereafter.

### Ascertainment of injury

By linking to the Patient Register and the Causes of Death Register, we identified the first injury resulting in healthcare visit (either as the primary or secondary diagnosis) or death (either as underlying or contributory cause) experienced by participants during follow-up. We also identified first injury event due to suicidal behavior (i.e., completed suicide and suicide attempt), accidents, assaults, and undetermined injury, where available information was insufficient to make a distinction between the aforementioned three subtypes, separately*.* Given our a priori hypotheses, we primarily focused on suicidal behavior and accidents in our main analysis. We further sub-grouped accidents into (1) falls, (2) transportation and accidents by other external factors, (3) accidents by natural forces or contact with animals or plants, (4) cutting or piercing, (5) poisoning, and (6) others (supplementary Table S1). Injuries that occurred before the start of the follow-up were defined as “history of injury” and not treated as outcomes; only the first injury occurring during the follow-up was registered as an outcome event.

The Patient Register covers >90% of hospital discharges nationwide for injury since 1987 and has a high validity (94.8%) for diagnosing injury [23]. Ascertainment of injury-related deaths in the Causes of Death Register is considered highly accurate [26] and complete [27].

### Covariates

Information about year of birth and region of residence was obtained from the Swedish Population and Housing Census in 1990, while information on the highest educational level was retrieved from the Swedish Education Register, which is updated through 2001. Comorbid psychiatric diagnoses were identified from the Patient Register from 1981 onward. PMD are commonly comorbid with psychiatric disorders, which may both mediate and confound the association between PMD and injury. We therefore defined psychiatric comorbidities for all women by identifying any diagnosis of psychiatric disorders (290-319 in ICD-8/9, F10-F90 in ICD-10) throughout follow-up from the Patient Register. Psychiatric comorbidities were then treated as a time-varying covariate.

### Statistical analysis

First, we compared the distributions of demographic characteristics between the reference and PMD groups in both the population analysis and the sibling analysis. The characteristics of PMD patients identified via clinical diagnosis and through treatment indication were evaluated separately. Next, we calculated unadjusted incidence rates (number of events divided by accumulated person-years) of injury in both groups, separately.

In the population analysis, we used Cox proportional hazards regression (attained age as the underlying timescale) to estimate hazard ratios (HRs) and 95% confidence intervals (CIs) of injury among women with PMD, compared to the reference group. We accounted for the relatedness of individuals (i.e., sisters) using robust sandwich estimator of variance in the population analysis [28]. The proportional hazard (PH) assumption was tested by plotting the Schoenfeld residuals. Additionally, we conducted a sibling analysis using stratified Cox proportional hazards regression. The sibling analysis contrasts the rates within each set of full sister discordant on PMD diagnosis and inherently controls for unmeasured factors (e.g., shared genetic and familial environmental factors) shared between sisters [29].

We performed separate analyses for the 4 types of injury: (1) suicidal behavior, (2) accidents, (3) assaults, and (4) undetermined injury. We further conducted an analysis of accident subtypes.

We conducted a series of sensitivity analyses to assess the robustness of the PMD classification and assess the potential impact of misclassification. To assess potential differences in outcomes among PMD patients by diagnosis type, we differentiated between PMD identified by (1) clinical diagnosis, (2) treatment indication, and (3) clinical diagnosis and treatment indication. Although all diagnoses in the Patient Register are made by specialists with high validity [23], the clinical diagnosis of PMD has not been validated. To further test the validity of PMD diagnoses, we restricted the PMD group to patients with at least two consecutive PMD diagnoses appearing at least 28 days apart in the Patient Register. We also conducted a simulation analysis and calculated hazard ratios for injury in testing conditions where 10–80% of PMD diagnoses were false positives [30]. Finally, because use of antidepressants or hormonal contraceptives may be associated with different risk of suicidal behavior [31, 32], a sensitivity analysis was conducted by restricting our primary analysis to untreated PMD.

In all analyses, attained age was used as the underlying timescale. We adjusted for calendar year of birth, educational level, region of residence, and history of injury in Model 1. Psychiatric comorbidities were additionally adjusted for in a time-varying manner in Model 2. However, we only employed Model 2 for the analyses subsequent to the primary analysis, whenever applicable. All covariates were categorized (Table 1).

**Table 1:** Characteristics of women with and without premenstrual disorders (PMD)

	Population cohort		Sibling cohort ^**a**^	
	Reference ***N*** (%)	PMD ***N*** (%)	Reference ***N*** (%)	PMD ***N*** (%)
**Total number**	1,472,310	18,628	631,236	7,674
**Year of birth**				
1960–1964	239,959 (16.3)	4346 (23.3)	80,813 (12.8)	1471 (19.2)
1965–1970	255,554 (17.4)	5080 (27.3)	111,933 (17.7)	2142 (27.9)
1970–1974	243,458 (16.5)	4097 (22.0)	112,549 (17.8)	1837 (23.9)
1975–1979	218,052 (14.8)	2428 (13.0)	103,626 (16.4)	1097 (14.3)
1980–1984	216,518 (14.7)	1628 (8.7)	106,187 (16.8)	753 (9.8)
1985–1990	298,769 (20.3)	1049 (5.6)	116,128 (18.4)	374 (4.9)
**Educational level**				
Primary	226,819 (15.4)	2304 (12.4)	104,799 (16.6)	957 (12.5)
High school	604,802 (41.1)	9435 (50.6)	262,378 (41.6)	3910 (51.0)
College and beyond	379,324 (25.8)	6005 (32.2)	166,447 (26.4)	2508 (32.7)
Unknown	261,365 (17.8)	884 (4.7)	97,612 (15.5)	299 (3.9)
**Region of residency**				
South	342,159 (23.2)	3602 (19.3)	150,946 (23.9)	1514 (19.7)
Middle	825,202 (56.0)	11,293 (60.6)	347,545 (55.1)	4597 (59.9)
North	304,949 (20.7)	3733 (20.0)	132,745 (21.0)	1563 (20.4)
**History of injury**				
No	1,327,241 (90.1)	14,169 (76.1)	571,236 (90.5)	5870 (76.5)
Yes	145,069 (9.9)	4459 (23.9)	60,000 (9.5)	1804 (23.5)
	**PYs (%)**	**PYs (%)**	**PYs (%)**	**PYs (%)**
**Psychiatric comorbidities**^**b**^				
No	13,820,488 (95.6)	83,418 (89.3)	6,050,657 (95.9)	34,195 (89.7)
Yes	636,709 (4.4)	10,005 (10.7)	261,787 (4.1)	3932 (10.3)

*N* number, *PYs* person-years

^a^Sibling cohort was restricted to women with at least one identifiable full sister in the population cohort

^b^Psychiatric disorder was considered as time-varying, i.e., person-years from the cohort entry if any diagnosis of psychiatric disorders occurred before cohort entry, or person-years from the date of diagnosis if any diagnosis of psychiatric disorders occurred during the follow-up

Because PMD has been associated with multiple psychiatric comorbidities [33], we repeated our primary analysis by separately adjusting for the number of psychiatric comorbidities and individual type of psychiatric comorbidities (e.g., substance abuse, schizophasia) in a time-varying way. Finally, as previous suicidal behavior is a strong predictor of suicidal behavior [34], we additionally adjusted for history of suicidal behavior when assessing the association between PMD and suicidal behavior.

To provide insights into the temporal pattern, for each PMD patient, we randomly selected 5 women from the study base who had not yet been diagnosed with PMD by the patient’s index date (i.e., the matching date); reference women were individually matched to PMD patients by birth year (within a 5-year range) and region of residency. We then plotted the cumulative incidence rates and CIs of suicidal behavior and accidents, from the diagnosis/matching date, using *survci* package in STATA. We further estimated HRs of suicidal behavior and accidents across different time windows, including within 1 year, >1 to 2 years, and >2 years after PMD diagnosis, separately.

A diagnosis of PMD may be delayed for years from symptom onset [35]. We therefore conducted an additional analysis to estimate the risk of injury preceding diagnosis among PMD patients (i.e., from cohort entry to the date of diagnosis), compared to women without PMD. Due to risk of a delay in PMD diagnosis, we also conducted a sensitivity analysis by excluding person-time preceding the diagnosis of PMD (i.e., from cohort entry to the date of diagnosis).

To shed light on the impact of psychiatric comorbidities, we performed an analysis stratified by psychiatric comorbidities by estimating HRs in the presence and absence of psychiatric comorbidities. To identify potential risk modifiers, we also performed stratified analyses for any injury by calendar year of birth, educational level, region of residency, and history of injury.

Data were prepared in SAS statistical software version 9.4 (SAS Institute, Cary, NC) and analyzed in Stata 15.1 (STATA, College Station, TX). The statistical significance was set at the nominal two-sided 5% level.

## Results

### Characteristics

In total, we identified 18,628 women with a clinical indication of PMD, of which 7674 had identifiable full sisters. The average age at diagnosis was 36 years (standard deviation 7 years). Compared with the general female population, women with PMD were older, had higher educational attainment, and were more likely to have a history of injury and psychiatric comorbidities (Table 1) at baseline. Similar patterns were observed in the sibling analysis. PMD patients identified from the Patient Register were slightly different from those identified from the Prescribed Drug Register (Supplementary Table S2).

### Risk of injury

**Fig. 1:**
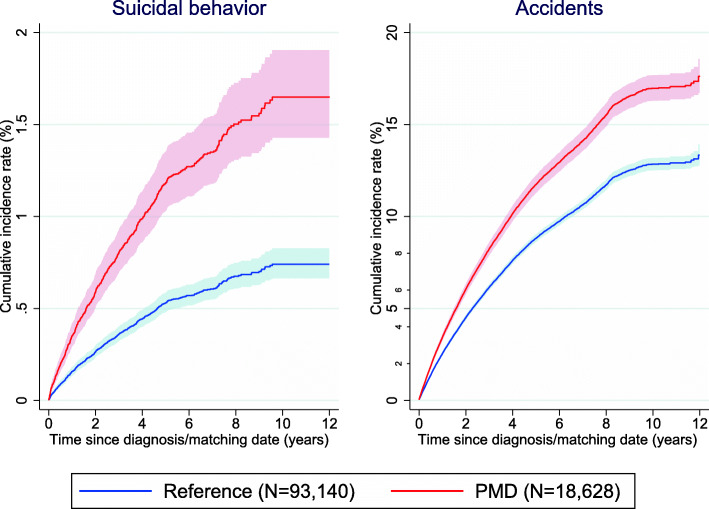
Cumulative incidence rates (CIR) of suicidal behavior and accidents. Each PMD patient was individually matched to 5 individuals without PMD at the diagnosis/matching date by birth year (within a 5-year range) and region of residency as the reference group

During the maximal follow-up of 12 years (mean 9.55 years), we identified a total of 22,636 women with injury due to suicidal behavior (216 in the PMD group), and 315,162 women with injury due to accidents (2191 in the PMD group) in the population analysis; of these, 9000 women with suicidal behavior (89 in the PMD group), and 132,900 women with accidents (895 in the PMD group) were included in the sibling analysis. After receiving a diagnosis of PMD, the cumulative incidence rate of suicidal behaviors and accidents rapidly increased over time and reached about 1.6% and 17%, respectively, by 12 years of follow-up, which were considerably higher than in the matched reference group (Fig. 1).

Compared to women without PMD, women with PMD were at increased risk of suicidal behavior (HR 1.65, 95% CI 1.43 to 1.89) and accidents (HR 1.28, 95% CI 1.23 to 1.34), in the population analysis. The sibling analysis gave similar estimates for accidents, though slightly lower estimates for suicidal behavior events with lower precision. Additional adjustment for psychiatric comorbidities minimally altered the associations with any injury and accidents in both population and sibling analyses. The association with suicidal behavior was considerably attenuated to non-significance in the sibling analysis (Table 2). For assaults and other undetermined injury, positive associations were observed in the population analysis but attenuated towards null in the sibling analysis (supplementary Table S3). We found elevated risks for all types of accidents among women with PMD in the population and similar associations in the sibling analysis (supplementary Table S4).

**Table 2:** Associations of premenstrual disorders (PMD) with subsequent risk of injury

	Population analysis						Sibling analysis ^**a**^					
	Reference	PMD	Model 1	***P***	Model 2	***P***	Reference	PMD	Model 1	***P***	Model 2	***P***
	***N*** (IR)	***N*** (IR)	HR (95% CI) ^***b***^		HR (95% CI) ^***c***^		***N*** (IR)	***N*** (IR)	HR (95% CI) ^***b***^		HR (95% CI) ^**c**^	
**Any injury**	336,632 (23.3)	2,390 (25.6)	1.37 (1.31–1.42)	<0.01	1.30 (1.25–1.36)	<0.01	141,677 (22.4)	973 (25.5)	1.31 (1.16–1.48)	<0.01	1.28 (1.14–1.45)	<0.01
Suicidal behavior	22,420 (1.3)	216 (2.1)	2.26 (1.97–2.59)	<0.01	1.65 (1.43–1.89)	<0.01	8,911 (1.2)	89 (2.1)	1.86 (1.21–2.87)	0.01	1.44 (0.87–2.40)	0.16
Accidents	312,971 (21.4)	2,191 (23.2)	1.32 (1.27–1.38)	<0.01	1.28 (1.23–1.34)	<0.01	132,005 (20.7)	895 (23.2)	1.29 (1.14–1.46)	<0.01	1.27 (1.12–1.44)	<0.01

*N* number, *IR* crude incidence rate per 1000 person-years, *HR* hazard ratio, *CI* confidence interval

^a^Analyses were stratified on full sister sets

^b^HRs were controlled for attained age as the underlying timescale, calendar year of birth (1960–1965, 1966–1970, 1971–1975, 1976–1980, 1981–1985, or 1986–1990), educational level (primary school, high school, college and beyond, or unknown), region of residence (south, middle, or north of Sweden), and history of injury (yes or no)

^c^HRs were additionally adjusted for psychiatric comorbidities (yes or no)

### Sensitivity analyses

Results stayed substantially similar to the main analysis when (1) adjusting for the number of psychiatric disorders or type-specific psychiatric comorbidities separately (supplementary Tables S5, S6); (2) adjusting for history of suicidal behavior when assessing the risk of suicidal behavior (supplementary Table S7); (3) restricting to PMD patients who received one or two clinical diagnoses made by specialists, regardless of the prescribed medications (supplementary Table S8); or (4) excluding the person-time preceding the diagnosis among PMD patients (supplementary Table S9).

Women with PMD who were not using antidepressants or oral contraceptives, were at significantly higher risk of suicidal behavior and accidents, in both population and sibling analyses (supplementary Table S10). Simulation analysis indicated that the observed associations in the population analysis remained up to the point where 80% of PMD diagnoses would be false positives (supplementary Table S11).

### Temporal pattern

**Fig. 2:**
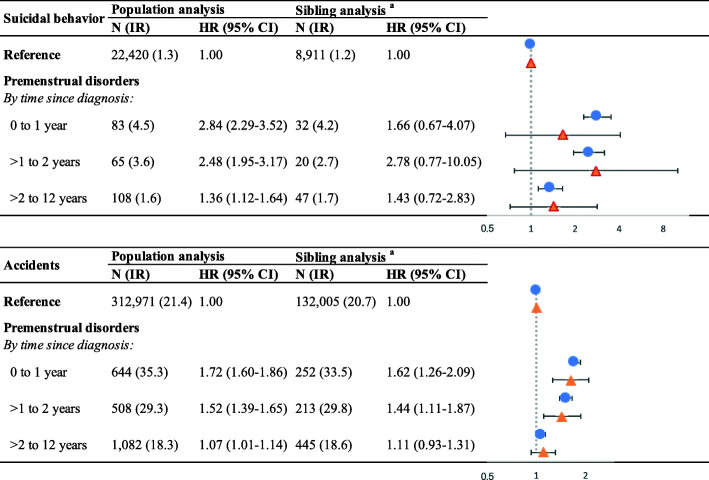
Hazard ratios (HRs) and 95% confidence intervals (CIs) of injury after receiving a diagnosis of premenstrual disorders (PMD). Sibling analysis was stratified on full sister sets. HRs were controlled for attained age as the underlying timescale and additionally adjusted for calendar year of birth (1960–1965, 1966–1970, 1971–1975, 1976–1980, 1981–1985, or 1986–1990), educational level (primary school, high school, college and beyond, or unknown), region of residence (south, middle, or north of Sweden), history of injury (yes or no), and psychiatric comorbidities (yes or no). Blue dots denote HRs estimated from the population analysis, while red triangles represent HRs estimated from the sibling analysis. N, number; IR, crude incidence rate per 1000 person-years; HR, hazard ratio; CI, confidence interval

The higher risk of suicidal behavior and accidents associated with PMD was strong within the first 2 years after PMD diagnosis, particularly the first year (HR 2.84, 95% CI 2.29 to 3.52 for suicidal behavior; HR 1.72, 95% CI 1.60 to 1.86 for accidents in the population analysis, Fig. 2). Relative risks declined somewhat by 2 years after PMD diagnosis (HR 1.43, 95% CI 1.12 to 1.64 for suicidal behavior and HR 1.07, 95% CI 1.01 to 1.14 for accidents in the population analysis). Similar results were observed in sibling analysis but not statistically significant for suicidal behavior due to small number of events. The risk of injury was already somewhat higher for PMD patients in the period pre-diagnosis (suicidal behavior: HR 1.67, 95% CI 1.50 to 1.86 in the population analysis; HR 1.43, 95% CI 0.96 to 2.14 in the sibling analysis; and accidents: HR 1.12, 95% CI 1.08 to 1.16 in the population analysis; HR 1.10, 95% CI 0.99 to 1.23 in the sibling analysis).

### Stratified analysis

In a stratified analysis, an increased risk of accidents was noted for women with PMD with and without psychiatric comorbidities in the population analysis, with slightly stronger association for accidents for PMD patients without psychiatric comorbidities (P for interaction=0.007; Table 3). However, this pattern was not observed in the sibling analysis (*P* for interaction=0.783). Comparable associations were observed for suicidal behavior in both population and sibling analyses. An increased risk of any injury was observed for women with PMD regardless of history of injury. The association was stronger for PMD with a history of injury in the population analysis, although there was lack of precision in the sibling analysis. Finally, year of birth, educational level, and region of residency did not clearly modify the associations with injury risk (supplementary Table S12).

**Table 3:** Associations of premenstrual disorders (PMD) with subsequent risk of injury, stratified by psychiatric comorbidities

	Population analysis				Sibling analysis ^a^			
	Reference ***N*** (IR)	PMD ***N*** (IR)	HR (95% CI) ^**b**^	***P*** for interaction	Reference ***N*** (IR)	PMD ***N*** (IR)	HR (95% CI) ^**b**^	***P*** for interaction
**Any injury**								
*By psychiatric comorbidities*								
No	306,980 (22.2)	1923 (23.1)	1.34 (1.28–1.40)	0.010	130,121 (21.5)	795 (23.2)	1.25 (1.08–1.41)	0.304
Yes	29,652 (46.6)	467 (46.7)	1.17 (1.06–1.28)		11,556 (44.1)	178 (45.3)	1.54 (1.01–2.07)	
**Suicidal behavior**								
*By psychiatric comorbidities*								
No	12,678 (0.8)	70 (0.8)	1.76 (1.34–2.19)	0.437	5156 (0.7)	28 (0.7)	1.41 (0.48–2.34)	0.870
Yes	9742 (11.8)	146 (12.3)	1.60 (1.33–1.86)		3755 (11.1)	61 (13.3)	1.50 (0.29–2.70)	
**Accidents**								
*By psychiatric comorbidities*								
No	288,068 (20.7)	1821 (21.7)	1.32 (1.26–1.38)	0.007	122,255 (20.1)	754 (22.0)	1.26 (1.09–1.43)	0.783
Yes	24,903 (34.8)	370 (34.5)	1.12 (1.00–1.24)		9750 (33.2)	141 (33.4)	1.37 (0.89–1.84)	

*N* number, *IR* crude incidence rate per 1000 person-years, *HR* hazard ratio, *CI* confidence interval

^a^Analyses were stratified on full sister sets

^b^HRs were controlled for attained age as the underlying timescale and additionally adjusted for calendar year of birth (1960–1965, 1966–1970, 1971–1975, 1976–1980, 1981–1985, or 1986–1990), educational level (primary school, high school, college and beyond, or unknown), region of residence (south, middle, or north of Sweden), and history of injury (yes or no)

## Discussion

In this nationwide cohort study of around 1.5 million women, we found that women a clinical indication of PMD had increased risks of injury due to accidents and suicidal behavior compared with the general female population, particularly within the first 2 years after PMD diagnosis. The increased risk was also statistically significant for accidents when comparing women with PMD with their full sisters, regardless of psychiatric comorbidities. The association of PMD with risk of suicidal behavior attenuated to non-significance in the sibling analysis after controlling for psychiatric comorbidities, suggesting a considerable contribution of concurrent psychiatric disorders.

Our findings are supported by a few earlier studies reporting a positive correlation between PMD and suicidal ideation [12], whereas a positive, independent association with suicidal attempts has only been previously noted for PMDD [13, 36]. The existing literature is limited to cross-sectional or retrospective case-control study designs, and therefore not informative as to the causal direction of the association of PMD with suicidal behavior. With prospectively collected data on both PMD and suicide behavior, the present study is the first to report an increased risk of suicidal behavior among women with PMD. Although somewhat attenuated and lacking power in our primary analysis, the results from the sibling analysis lend some support to these findings. Indeed, psychological distress, a consistent predictor of suicidal behavior [37], is common among women with PMD due to the cyclic premenstrual symptoms and reduced quality of life [9] and may predispose them to higher risk of suicidal behavior.

No previous study has systematically investigated the association between PMD and accidents in a population-based setting. Two small retrospective studies found a positive correlation between premenstrual symptoms and accidents in hospitalized patients and female soccer players [21, 22]. Our findings therefore add to the knowledge base that, compared to the general population, women with clinically diagnosed/indicated PMD are at higher risk of accidents overall (experienced by approximately 17% of PMD patients over the clinical course in our sample) and for specific types of accidents. This may be explained by the negative influence of premenstrual symptoms on energy, concentration, neuromuscular coordination, and perception. It has been documented that PMD patients have impaired manual dexterity during the premenstrual phase [38] and poorer concentration and coordination [39], which might contribute to increased falls and transportation accidents. Moreover, PMD patients often suffer from emotional problems (e.g., outbursts of anger and irritation) before menstruation, which might increase their risk of accidents [40]. Yet, further research is needed to understand the underlying mechanisms between PMD and risk for accidents.

Previous studies have shown that psychiatric comorbidities are common among women with PMD. Approximately 40% of cases suffer comorbid depressive symptoms and 70% suffer comorbid anxiety symptoms [13], both of which are well-known to be risk factors for suicide [41]. Indeed, in our study, the additional adjustment for the presence and/or number of psychiatric comorbidities attenuated the observed associations for suicidal behavior, which is consistent with previous findings [13] and suggest considerable contribution of psychiatric comorbidities to the studied association. However, psychiatric comorbidities can confound and/or mediate the studied association. For instance, PMD can prospectively lead to the development of major depression [42], which results in an episode of suicidal behavior [43]. In this scenario, depression is a mediator to the effect of PMD on suicidal behavior and adjusting for mediators would lead to conservative results [44]. It is also possible that depression leads to both the development of PMD and suicidal behavior, acting as a confounder. However, we have observed largely comparable associations, although the CIs are wide, among women with and without psychiatric comorbidities, lending support to the notion that the observed association may not be entirely explained by psychiatric comorbidities.

Our study shows that the increased risk of injury, either due to suicidal behavior or accidents, was most prominent within 2 years after diagnosis. This may reflect the severity of symptoms leading to diagnosis, as it has been shown that it can take more than 5 years on average between symptom onset and PMS diagnosis [35]. Moreover, the increased risk immediately following a diagnosis of severe illness has been noted for many diseases and is likely due the increased stress associated with living with the disease and/or undergoing intensive treatment [37]. Future research is needed to understand patients’ perception of PMD diagnosis and follow-up. The attenuation of this elevated risk 2 years post-diagnosis may be explained by the efficacy of antidepressants [45, 46] or oral contraceptives [38, 47] treatments for PMD. Indeed, additional analysis restricted to women untreated for PMD revealed a robust association with both suicidal behavior and accidents in both population and sibling analyses. However, it is notable that risk remained elevated for 12 years after PMD diagnosis. Additionally, our data indicate that the elevated risk of injury also precedes PMD diagnosis, which might be due to a delay in diagnosing women with these conditions [35].

In addition to the overall elevated risk of injury among women with PMD, the risk of injury may vary among PMD women between late luteal phase and symptom-free days. Yet, the chronic and cyclic PMD symptoms and resulting impairments in interpersonal relationships and social activities may have a lasting impact on women with PMD. Indeed, several cross-sectional studies have indicated that PMD are correlated with lower quality of life in daily lives in both mental and physical domains [39, 48–50], and a clinical trial has noted impaired quality of life even during symptom-free days (i.e., in the follicular phase), compared to healthy women [9]. Moreover, women with PMD commonly have comorbid depression and anxiety [33] resulting in lasting mental distress beyond the late luteal phase. Our data indeed indicate that psychiatric comorbidities may at least partly explain the association between PMD and suicidal behavior suggesting the impact of PMD may last beyond the late luteal phase. However, future research is needed to examine the potentially varying risk of injury across the menstrual cycle.

### Strength and limitations

The major merit of our study is the large-scale population-based cohort with prospectively collected information on both PMD with clinical indications and injury, which minimizes bias due to selection and measurement. The sibling analysis further allows us to rigorously control for familial factors shared between full sisters, such as genetic factors and familial environment during childhood. However, our study also has limitations. First, the clinical diagnoses of PMD have not been validated in the Swedish Patient Register. The healthcare guidelines in most regions in Sweden, for instance the one in Stockholm [24], have specified that “the diagnosis must be confirmed by the woman making daily prospective evaluation of symptoms for at least two consecutive menstrual cycles”. The clinical guidelines are often well followed in the state-funded public healthcare system in Sweden. Furthermore, the quality for the diagnoses in the Patient Register has been considered fairly high in general [51] and for a range of psychiatric disorders [52–55] and OB/GYN diseases [23, 56]. Moreover, our sensitivity analysis limited to PMD with two consecutive diagnoses 28 days apart have showed highly similar results to the primary population analysis, although we lacked power in the sibling analysis. In addition, our simulation analysis indicates that the observed association between PMD and injury in the population analysis would remain unless >80% of PMD diagnoses were incorrect. In addition, PMD identified only via treatment indication from the Prescribed Drug Register, and not clinical diagnosis, might represent less severe PMD. However, our sensitivity analysis restricted to PMD diagnosed in specialist care (as obtained from the Patient Register) yielded very similar results. Second, it might take years and many healthcare visits from the time of symptom onset to the receipt of a clinical diagnosis of PMD [35]. Thus, the date of PMD diagnosis in our data may not reflect either the timing of symptom onset or first clinical diagnosis, particularly for women who diagnosed before 2001 (i.e., when the Patient Register started to collect specialists-based outpatient diagnoses). This is supported by the higher proportion of PMD women with history of injury at cohort entry as well as the increased risk of injury before the date of diagnosis reported during the observation period. However, a sensitivity analysis excluding person-time preceding diagnosis among PMD patients yielded highly similar results. Third, the identified PMD individuals likely represent a patient group with most severe PMD symptomology, particularly those who felt obliged to seek help from healthcare and those who had more severe psychiatric comorbidities. Our findings may therefore not be generalized to individuals with milder premenstrual symptoms. Fourth, we lacked information on some potential confounding factors, e.g., childhood abuse [57, 58], smoking [59, 60], and had considerable missing data on educational level. However, we observed robust associations between PMD and accidents in the sibling analysis, which controls for factors shared between full sisters. Lastly, it is possible that women with other patterns of menstruation-related symptoms, such as premenstrual exacerbation of a pre-existing psychiatric disorder, are also at increased risk of injury. Future research is therefore needed for other menstrual cycle-related disorders [61]*.*

## Conclusions

Our findings suggest that women with a clinical indication of PMD are at increased risks of injury, particularly due to accidents. Health professionals may consider higher vigilance for such risk in women with clinically diagnosed PMD, particularly within the first 2 years after diagnosis. Future research is needed to understand the risk of injury among women with milder premenstrual symptoms.

## Supplementary Information

**Additional file 1: Supplementary Table S1.** Identification codes for premenstrual disorders and injuries in the Registers. **Supplementary Table S2.** Characteristics of women with premenstrual disorders (PMD) identified from the Patient Register and the Prescribed Drug Register. **Supplementary Table S3.** Associations of premenstrual disorders (PMD) with subsequent risks of assaults and other injuries. **Supplementary Table S4.** Associations of premenstrual disorders (PMD) with subsequent risks of different subtypes of accidents. **Supplementary Table S5.** Associations of premenstrual disorders (PMD) with subsequent risk of injury, with adjustment for binary psychiatric cormorbidities (yes or no) vs. number of psychiatric comorbidities. **Supplementary Table S6.** Associations of premenstrual disorders (PMD) with subsequent risk of injury, with adjustment for any vs. specific psychiatric disorder. **Supplementary Table S7.** Associations of premenstrual disorders (PMD) with subsequent risk of suicidal behavior: comparing adjustment for history of injury with adjustment for history of suicidal behavior. **Supplementary Table S8.** Associations of premenstrual disorders (PMD) with subsequent risk of injury by restricting to PMD with a clinical diagnosis, a treatment indication, both diagnosis and indication, and at least two consecutive clinical diagnoses. **Supplementary Table S9.** Associations of premenstrual disorders (PMD) with subsequent risk of injury by excluding the person-time preceding the diagnosis among PMD cases. **Supplementary Table S10.** Associations of premenstrual disorders (PMD) with subsequent risk of injury by restricting to untreated specialist-diagnosed PMD. **Supplementary Table S11.** Associations of premenstrual disorders (PMD) with subsequent risk of injury in the population analysis: a simulation analysis by randomly assigning a proportion of PMD diagnoses as false positive. **Supplementary Table S12.** Associations of premenstrual disorders (PMD) with subsequent risk of injury, stratified by year of birth, educational level, region of residency, and history of injury.

## Data Availability

Data are from the Swedish Population and Housing Census, Causes of Death Register, Migration Register, Swedish Multi-generation Register, Patient Register, Prescribed Drug Register, and Swedish Education Register. Data cannot be put into a public data repository due to Swedish law but are available by applying through Statistics Sweden or the Swedish National Board of Health and Welfare. Detailed information on data application can be found in the following links: https://www.scb.se/vara-tjanster/bestalla-mikrodata/ and https://bestalladata.socialstyrelsen.se/.

## Abbreviations

HRs: Hazard ratios
CIs: Confidence intervals
PMD: Premenstrual disorders
PMS: Premenstrual syndrome
PMDD: Premenstrual dysphoric disorder
ACOG: The American College of Obstetricians and Gynecologists
DSM: The Diagnostic and Statistical Manual of Mental Disorders
ICD: International Classification of Diseases
ATC: The Anatomical Therapeutic Chemical
PH: Proportional hazard
SSRIs: Selective serotonin reuptake inhibitors
