# Supplementary material for: Clinical indications of premenstrual disorders and subsequent risk of injury: a population-based cohort study in Sweden
Source: BMC Med. 2021 May 26;19:119. doi: 10.1186/s12916-021-01989-4 (PMC8152351; doi:10.1186/s12916-021-01989-4)
Supplement: Supplementary file 1 — Additional file 1: Supplementary Table S1. Identification codes for premenstrual disorders and injuries in the Registers. Supplementary Table S2. Characteristics of women with premenstrual disorders (PMD) identified from the Patient Register and the Prescribed Drug Register. Supplementary Table S3. Associations of premenstrual disorders (PMD) with subsequent risks of assaults and other injuries. Supplementary Table S4. Associations of premenstrual disorders (PMD) with subsequent risks of different subtypes of accidents. Supplementary Table S5. Associations of premenstrual disorders (PMD) with subsequent risk of injury, with adjustment for binary psychiatric cormorbidities (yes or no) vs. number of psychiatric comorbidities. Supplementary Table S6. Associations of premenstrual disorders (PMD) with subsequent risk of injury, with adjustment for any vs. specific psychiatric disorder. Supplementary Table S7. Associations of premenstrual disorders (PMD) with subsequent risk of suicidal behavior: comparing adjustment for history of injury with adjustment for history of suicidal behavior. Supplementary Table S8. Associations of premenstrual disorders (PMD) with subsequent risk of injury by restricting to PMD with a clinical diagnosis, a treatment indication, both diagnosis and indication, and at least two consecutive clinical diagnoses. Supplementary Table S9. Associations of premenstrual disorders (PMD) with subsequent risk of injury by excluding the person-time preceding the diagnosis among PMD cases. Supplementary Table S10. Associations of premenstrual disorders (PMD) with subsequent risk of injury by restricting to untreated specialist-diagnosed PMD. Supplementary Table S11. Associations of premenstrual disorders (PMD) with subsequent risk of injury in the population analysis: a simulation analysis by randomly assigning a proportion of PMD diagnoses as false positive. Supplementary Table S12. Associations of premenstrual disorders (PMD) with subsequent risk of injury, [file 12916_2021_1989_MOESM1_ESM.docx]

**Clinical Indications of Premenstrual Disorders and Subsequent Risk of Injury: A Population-Based Cohort Study in Sweden**

Qian Yang, M.D.^1^*, Arvid Sjölander, Ph.D^1^, Yuchen Li, M.D.^1^, Alexander Viktorin, Ph.D^1^, Elizabeth R. Bertone-Johnson, ScD^2, 3^, Weimin Ye, M.D., Ph.D^1^, Fang Fang, M.D., Ph.D^4^, Unnur A. Valdimarsdóttir, Ph.D^1, 5, 6†^, Donghao Lu, M.D., Ph.D ^4, 6†^

**Contents:**

**Supplementary Table S1 Identification codes for premenstrual disorders and injuries in the Registers.**

**Supplementary Table S2 Characteristics of women with premenstrual disorders (PMD) identified from the Patient Register and the Prescribed Drug Register.**

**Supplementary Table S3 Associations of premenstrual disorders (PMD) with subsequent risks of assaults and other injuries.**

**Supplementary Table S4 Associations of premenstrual disorders (PMD) with subsequent risks of different subtypes of accidents.**

**Supplementary Table S5 Associations of premenstrual disorders (PMD) with subsequent risk of injury, with adjustment for binary psychiatric cormorbidities (yes or no) vs. number of psychiatric comorbidities.**

**Supplementary Table S6 Associations of premenstrual disorders (PMD) with subsequent risk of injury, with adjustment for any vs. specific psychiatric disorder.**

**Supplementary Table S7 Associations of premenstrual disorders (PMD) with subsequent risk of suicidal behavior: comparing adjustment for history of injury with adjustment for history of suicidal behavior.**

**Supplementary Table S8 Associations of premenstrual disorders (PMD) with subsequent risk of injury by restricting to PMD with a clinical diagnosis, a treatment indication, both diagnosis and indication, and at least two consecutive clinical diagnoses.**

**Supplementary Table S9 Associations of premenstrual disorders (PMD) with subsequent risk of injury by excluding the person-time preceding the diagnosis among PMD cases.**

**Supplementary Table S10 Associations of premenstrual disorders (PMD) with subsequent risk of injury by restricting to untreated specialist-diagnosed PMD.**

**Supplementary Table S11 Associations of premenstrual disorders (PMD) with subsequent risk of injury in the population analysis: a simulation analysis by randomly assigning a proportion of PMD diagnoses as false positive.**

**Supplementary Table S12 Associations of premenstrual disorders (PMD) with subsequent risk of injury, stratified by year of birth, educational level, region of residency, and history of injury.**

**Supplementary Table S1 Identification codes for premenstrual disorders and injuries in the Registers**

| **Premenstrual disorders** | | |  |
| --- | --- | --- | --- |
|  |  |  | **Number** |
|  | **ICD-10** | **ICD-9** |  |
| **Patient Register** | N943 | 625E | 9,109 (48.9%) |
|  | **ATC** | **Key words ^a^** |  |
| **Prescribed Drug Register** |  |  | 9,519 (51.1%) |
| Anti-depressants | N06AB, N06AX, N06AA | Swedish: "PMS" , "PREMENSTRUELLT SYNDROM", "PREMENSTRUELLT DYSFORSIKT SYNDROM", "PREMENSTRUELLT DYSFORI", "PMD", "PMDD", "PMDS", "MENS" (for antidepressants)  English translations: PMS" , "PREMENSTRUAL SYNDROME", "PREMENSTRUAL DYSPHORIC SYNDROME", "PREMENSTRUAL DYSPHORIA", "PMD", "PMDD", "PMDS", "MENSES" (for antidepressants) | 9,249 (49.7%) |
| Oral contraceptives | G03A, G02B |  | 270 (1.4%) |
| **Injury ^b^** | | |  |
|  | **ICD-10** | **ICD-9** |  |
| Overall | V01-Y34, Y85-86, Y870-871 | E807-E869, E880-E929, E950-E969, E980-E989 |  |
| *By manner of Intent* |  |  |  |
| Accidents | V01-X59, Y85-Y86 | E800–E869, E880–E929 |  |
| Sucidal behavior | X60-X84, Y870 | E950–E959 |  |
| Assaults | X85-Y09, Y871 | E960–E969 |  |
| Other undetermined injuries | Y10-Y34 | E980-E989 |  |
| *Accident subtypes* |  |  |  |
| Fall | W00-W19, Y85-Y86 | E880–E886, E888 |  |
| Transportation or by other external forces | W20-W22, W50-W52, V01-V99 | E800-E848, E916–E917 |  |
| By natural forces, contacts with animals/plants | W53-W64, X20-X39 | E900–E901, E905-E909, E9208, E9209, E9262, E9288 |  |
| Cutting or piercing | W25-W29, W45, | E920 |  |
| Poisoning | X40-X49, Y352 | E850-E869 |  |
| Others | The remaining ICD codes | The remaining ICD codes |  |

ATC, the Anatomical Therapeutic Chemical Classification; ICD, International Classification of Diseases; Swedish revisions of the International Classification of Disease, 9th (1991-1996) and 10th (1997-2010).

^a^ Key words used for identification of premenstrual disorders in Prescribed Drug Register were developed by systematically reviewing prescriptions for premenstrual disorder diagnoses that were randomly selected from the Patient Register. Prescriptisons included key words as “DEMENS”, “KLIMAK” or “MENOPAU” were excluded as those prescriptions were prescribed for dementia, climacteric or menopause, respectively.

^b^ All injuries were identified by using E-codes from hospital discharge records in the Patient Register, and by using underlying and secondary causes from death certificates in the Cause of Death Register.

**Supplementary Table S2 Characteristics of women with premenstrual disorders (PMD) identified from the Patient Register and the Prescribed Drug Register.**

|  | **PMD from the Patient Register ^a^** | **PMD from the Prescribed Drug Register ^b^** |
| --- | --- | --- |
|  | **N (%)** | **N (%)** |
| **Total number** | (n=9,109) | (n=9,519) |
| **Year of birth** | | |
| 1960-1964 | 2,425 (26.6%) | 1,921 (20.2%) |
| 1965-1970 | 2,658 (29.2%) | 2,422 (25.4%) |
| 1970-1974 | 1,924 (21.1%) | 2,173 (22.8%) |
| 1975-1979 | 1,059 (11.6%) | 1,369 (14.4%) |
| 1980-1984 | 659 (7.2%) | 969 (10.2%) |
| 1985-1990 | 384 (4.2%) | 665 (7.0%) |
| **Educational level** | | |
| Primary | 1,180 (13.0%) | 1,124 (11.8%) |
| High school | 4,861 (53.4%) | 4,574 (48.1%) |
| College and beyond | 2,714 (29.8%) | 3,291 (34.6%) |
| Unknown | 354 (3.9%) | 530 (5.6%) |
| Region of residency | | |
| South | 2,125 (23.3%) | 1,477 (15.5%) |
| Middle | 5,029 (55.2%) | 6,264 (65.8%) |
| North | 1,955 (21.5%) | 1,778 (18.7%) |
| History of injury | | |
| No | 7,032 (77.2%) | 7,137 (75.0%) |
| Yes | 2,077 (22.8%) | 2,382 (25.0%) |
|  |  |  |
|  | **PYs (%)** | **PYs (%)** |
| **Psychiatric** **comorbidities ^c^** | | |
| No | 46,826 (87.8%) | 36,592 (91.3%) |
| Yes | 6,529 (12.2%) | 3,476 (8.7%) |

N, number; PYs, person-years.

^a^ PMD were identified with the ICD code from the Patient Register (codes: 625E in ICD-9 and N943 in ICD-10).

^b^ PMD were identified with indication from the Prescribed Drug Register.

^c^ Psychiatric disorder was considered as time-varying, i.e., person-years from the cohort entry if any diagnosis of psychiatric disorders occurred before cohort entry, or person-years from the date of diagnosis if any diagnosis of psychiatric disorders occurred during the follow-up period.

**Supplementary Table S3 Associations of premenstrual disorders (PMD) with subsequent risks of assaults and undertermined injuries.**

|  | **Population analysis** | | | | **Sibling analysis ^a^** | | | |
| --- | --- | --- | --- | --- | --- | --- | --- | --- |
|  | **Reference**  **N (IR)** | **PMD**  **N (IR)** | **Model 1**  **HR (95% CI) ^b^** | **Model 2**  **HR (95% CI) ^c^** | **Reference**  **N(IR)** | **PMD**  **N(IR)** | **Model 1**  **HR (95% CI) ^b^** | **Model 2**  **HR (95% CI) ^c^** |
| Assaults | 14,324 (0.9) | 96 (0.9) | 1.47 (1.20-1.81) | 1.24 (1.00-1.52) | 3,675 (0.5) | 22 (0.5) | 1.15 (0.64-2.04) | 1.06 (0.58-1.93) |
| Undertermined injuries | 10,547 (0.6) | 75 (0.7) | 1.39 (1.11-1.75) | 1.20 (0.95-1.51) | 4,441 (0.6) | 33 (0.8) | 1.04 (0.55-1.95) | 0.93 (0.47-1.81) |

N, number; IR, crude incidence rate per 1000 person-years; HR, hazard ratio; CI, confidence interval.

^a^ Analyses were stratified on full-sister sets.

^b^ HRs were controlled for attained age as the underlying timescale, calendar year of birth (1960-1965, 1966-1970, 1971-1975, 1976-1980, 1981-1985, or 1986-1990), educational level (primary school, high school, college and beyond, or unknown), region of residence (south, middle, or north of Sweden) and history of injury (yes or no).

^c^ HRs were additionally adjusted for psychiatric comorbidities (yes or no).

**Supplementary Table S4 Associations of premenstrual disorders (PMD) with subsequent risks of different subtypes of accidents.**

|  | **Population analysis** | | | **Sibling analysis ^a^** | | |
| --- | --- | --- | --- | --- | --- | --- |
|  | **Reference** | **PMD** | **HR (95% CI) ^b^** | **Reference** | **PMD** | **HR (95% CI) ^b^** |
|  | **N (IR)** | **N (IR)** |  | **N (IR)** | **N (IR)** |  |
| **Fall** | 137,961 (10.0) | 929 (10.2) | 1.20 (1.13-1.28) | 51,474 (9.3) | 323 (9.5) | 1.23 (1.00-1.50) |
| **Transportation or by other external forces** | 86,307 (6.4) | 517 (5.7) | 1.31 (1.20-1.43) | 31,167 (5.9) | 201 (6.2) | 1.25 (0.96-1.62) |
| **By natural forces contacts with animals/plants** | 20,923 (1.6) | 167 (1.9) | 1.38 (1.18-1.60) | 7,006 (1.4) | 51 (1.6) | 1.21 (0.76-1.93) |
| **Cutting or piercing** | 17,204 (1.3) | 131 (1.5) | 1.36 (1.14-1.62) | 6,038 (1.2) | 50 (1.6) | 1.11 (0.67-1.85) |
| **Poisoning** | 4,084 (0.3) | 37 (0.4) | 1.42 (1.02-1.98) | 1,334 (0.3) | 12 (0.4) | 2.14 (0.62-7.33) |
| **Others** | 46,492 (3.5) | 410 (4.6) | 1.38 (1.25-1.52) | 16,461 (3.2) | 142 (4.5) | 1.38 (1.02-1.86) |

N, number; IR, crude incidence rate per 1000 person-years; HR, hazard ratio; CI, confidence interval.

^a^ Analyses were stratified on full-sister sets.

^b^ HRs were controlled for attained age as the underlying timescale and additionally adjusted for calendar year of birth (1960-1965, 1966-1970, 1971-1975, 1976-1980, 1981-1985, or 1986-1990), educational level (primary school, high school, college and beyond, or unknown), region of residence (south, middle, or north of Sweden), history of injury (yes or no) and psychiatric comorbidities (yes or no).

**Supplementary Table S5 Associations of premenstrual disorders (PMD) with subsequent risk of injury, with adjustment for binary psychiatric cormorbidities (yes or no) vs. number of psychiatric comorbidities.**

|  | **Population analysis** | | | | **Sibling analysis ^a^** | | | |
| --- | --- | --- | --- | --- | --- | --- | --- | --- |
|  | **Reference**  **N (IR)** | **PMD**  **N (IR)** | **HR (95% CI) *^b^*** | **HR (95% CI) *^c^*** | **Reference**  **N (IR)** | **PMD**  **N (IR)** | **HR (95% CI) *^b^*** | **HR (95% CI) ^c^** |
| **Any injury** | 336,632 (23.3) | 2,390 (25.6) | 1.30 (1.25-1.36) | 1.30 (1.25-1.35) | 141,677 (22.4) | 973 (25.5) | 1.28 (1.14-1.45) | 1.28 (1.13-1.45) |
| Suicidal behavior | 22,420 (1.3) | 216 (2.1) | 1.65 (1.43-1.89) | 1.55 (1.35-1.77) | 8,911 (1.2) | 89 (2.1) | 1.44 (0.87-2.40) | 1.43 (0.85-2.40) |
| Accidents | 312,971 (21.4) | 2,191 (23.2) | 1.28 (1.23-1.34) | 1.28 (1.23-1.34) | 132,005 (20.7) | 895 (23.2) | 1.27 (1.12-1.44) | 1.27 (1.12-1.44) |

N, number; IR, crude incidence rate per 1000 person-years; HR, hazard ratio; CI, confidence interval.

^a^ Analyses were stratified on full-sister sets.

^b^ HRs were controlled for attained age as the underlying timescale, calendar year of birth (1960-1965, 1966-1970, 1971-1975, 1976-1980, 1981-1985, or 1986-1990), educational level (primary school, high school, college and beyond, or unknown), region of residence (south, middle, or north of Sweden), history of injury (yes or no) and ***psychiatric comorbidities (yes or no)***.

^c^ HRs were controlled for attained age as the underlying timescale, calendar year of birth (1960-1965, 1966-1970, 1971-1975, 1976-1980, 1981-1985, or 1986-1990), educational level (primary school, high school, college and beyond, or unknown), region of residence (south, middle, or north of Sweden), history of injury (yes or no) and ***the number of psychiatric comorbidities*** (0, 1, 2, ≥3 according to the 10-level subchapter categories defined in ICD-10 (e.g., codes F00-F09, F10-19, F20-29, etc.).

**Supplementary Table S6 Associations of premenstrual disorders (PMD) with subsequent risk of injury, with adjustment for any vs. specific psychiatric disorder.**

|  | **Any injury** | | **Suicidal behavior** | | **Accidents** | |
| --- | --- | --- | --- | --- | --- | --- |
|  | HR (95% CI) ^a^ | | HR (95% CI) ^a^ | | HR (95% CI) ^a^ | |
|  | Population analysis | Sibling analysis ^b^ | Population analysis | Sibling analysis ^b^ | Population analysis | Sibling analysis ^b^ |
| Adjusting for: |  |  |  |  |  |  |
| Any psychiatric comorbidities | 1.30 (1.25-1.36) | 1.28 (1.14-1.45) | 1.65 (1.43-1.89) | 1.44 (0.87-2.40) | 1.28 (1.23-1.34) | 1.27 (1.12-1.44) |
| Substance abuse | 1.35 (1.29-1.40) | 1.31 (1.16-1.48) | 2.07 (1.81-2.37) | 1.81 (1.15-2.84) | 1.31 (1.26-1.37) | 1.29 (1.13-1.46) |
| Schizophenia | 1.37 (1.31-1.42) | 1.31 (1.16-1.48) | 2.21 (1.93-2.53) | 1.90 (1.22-2.96) | 1.32 (1.27-1.38) | 1.29 (1.14-1.46) |
| Mood disorders | 1.33 (1.27-1.38) | 1.29 (1.15-1.46) | 1.64 (1.43-1.88) | 1.85 (1.15-2.97) | 1.30 (1.25-1.36) | 1.28 (1.13-1.45) |
| Neurotic disorders | 1.33 (1.28-1.39) | 1.29 (1.15-1.46) | 1.85 (1.62-2.12) | 1.52 (0.96-2.42) | 1.30 (1.25-1.36) | 1.28 (1.13-1.45) |
| Eating disorders | 1.36 (1.31-1.42) | 1.31 (1.16-1.48) | 2.18 (1.90-2.49) | 1.88 (1.22-2.90) | 1.32 (1.26-1.37) | 1.29 (1.14-1.46) |
| Personality disorders | 1.36 (1.30-1.41) | 1.31 (1.16-1.48) | 2.06 (1.80-2.36) | 1.87 (1.21-2.89) | 1.32 (1.26-1.37) | 1.29 (1.14-1.46) |
| Others | 1.36 (1.31-1.42) | 1.31 (1.16-1.48) | 2.17 (1.89-2.48) | 1.92 (1.24-2.97) | 1.32 (1.26-1.37) | 1.29 (1.14-1.46) |

N, number; IR, crude incidence rate per 1000 person-years; HR, hazard ratio; CI, confidence interval.

^a^ HRs were controlled for attained age as the underlying timescale, calendar year of birth (1960-1965, 1966-1970, 1971-1975, 1976-1980, 1981-1985, or 1986-1990), educational level (primary school, high school, college and beyond, or unknown), region of residence (south, middle, or north of Sweden), history of injury (yes or no), and any psychiatric comorbidities (yes or no)/index psychiatric comorbidities (yes or no).

^b^ Analyses were stratified on full-sister sets.

**Supplementary Table S7 Associations of premenstrual disorders (PMD) with subsequent risk of suicidal behavior: comparing adjustment for history of injury with adjustment for history of suicidal behavior.**

|  | **Population analysis** | | | | **Sibling analysis ^a^** | | | |
| --- | --- | --- | --- | --- | --- | --- | --- | --- |
|  | Reference | PMD | HR (95% CI) *^b^* | HR (95% CI) *^c^* | Reference | PMD | HR (95% CI) *^b^* | HR (95% CI) ^c^ |
| Suicidal behavior | 22,420 (1.3) | 216 (2.2) | 1.65 (1.43-1.89) | 1.66 (1.45-1.90) | 8,911 (1.2) | 89 (2.1) | 1.44 (0.87-2.40) | 1.46 (0.87-2.44) |

N, number; IR, crude incidence rate per 1000 person-years; HR, hazard ratio; CI, confidence interval.

^a^ Analyses were stratified on full-sister sets.

^b^ HRs were controlled for attained age as the underlying timescale, calendar year of birth (1960-1965, 1966-1970, 1971-1975, 1976-1980, 1981-1985, or 1986-1990), educational level (primary school, high school, college and beyond, or unknown), region of residence (south, middle, or north of Sweden), ***history of injury (yes or no)*** and psychiatric comorbidities (yes or no).

^c^ HRs were controlled for attained age as the underlying timescale, calendar year of birth (1960-1965, 1966-1970, 1971-1975, 1976-1980, 1981-1985, or 1986-1990), educational level (primary school, high school, college and beyond, or unknown), region of residence (south, middle, or north of Sweden), ***history of suicidal behavior (yes or no)*** and psychiatric comorbidities (yes or no).

**Supplementary Table S8 Associations of premenstrual disorders (PMD) with subsequent risk of injury by restricting to PMD with a clinical diagnosis, a treatment indication, both diagnosis and indication, and at least two consecutive clinical diagnoses.**

|  | **Population analysis** | | | **Sibling analysis ^a^** | | |
| --- | --- | --- | --- | --- | --- | --- |
|  | **Reference**  **N (IR)** | **PMD**  **N (IR)** | **HR (95% CI)  ^b^** | **Reference**  **N (IR)** | **PMD**  **N (IR)** | **HR (95% CI) ^b^** |
| **Restricting to women with clinical diagnosis of PMD ascertained from the Patient Register** | | | | | | |
| **Any injury** | 336,632 (23.3) | 1,628 (30.5) | 1.47 (1.40-1.55) | 141,006 (22.4) | 652 (30.7) | 1.39 (1.19-1.62) |
| Suicidal behavior | 22,420 (1.3) | 161 (2.6) | 1.84 (1.57-2.15) | 8,864 (1.2) | 65 (2.7) | 1.99 (1.06-3.73) |
| Accidents | 312,971 (21.4) | 1,491 (27.5) | 1.45 (1.38-1.53) | 131,385 (20.7) | 597 (27.6) | 1.35 (1.16-1.58) |
| **Restricting to women with clinical indication of PMD ascertained from the Prescribed Drug Register** | | | | | | |
| **Any injury** | 336,632 (23.3) | 762 (19.0) | 1.04 (0.97-1.12) | 141,035 (22.4) | 314 (19.1) | 1.10 (0.89-1.35) |
| Suicidal behavior | 22,420 (1.3) | 55 (1.3) | 1.27 (0.97-1.66) | 8,873 (1.2) | 23 (1.3) | 0.70 (0.28-1.77) |
| Accidents | 312,971 (21.4) | 700 (17.4) | 1.03 (0.95-1.11) | 131,413 (20.7) | 292 (17.7) | 1.14 (0.92-1.41) |
| **Restricting to women with clinical diagnosis and indication of PMD identifiable from both the Patient Register and the Prescribed Drug Register ^c^** | | | | | | |
| **Any injury** | 336,632 (23.3) | 355 (27.9) | 1.45 (1.31-1.61) | 140,540 (22.5) | 159 (29.9) | 1.53 (1.14-2.07) |
| Suicidal behavior | 22,420 (1.3) | 27 (1.9) | 1.72 (1.18-2.50) | 8,837 (1.2) | 11 (1.8) | 1.38 (0.44-4.36) |
| Accidents | 312,971 (21.4) | 322 (25.1) | 1.41 (1.26-1.57) | 130,955 (20.7) | 148 (27.6) | 1.66 (1.21-2.27) |
| **Restricting to women with at least two clinical diagnoses of PMD in two menstrual cycles ascertained from the Patient Register ^d^** | | | | | | |
| Any injury | 336,632 (23.3) | 286 (27.7) | 1.31 (1.16-1.47) | 140,499 (22.5) | 131 (31.6) | 1.24 (0.89-1.73) |
| Suicidal behavior | 22,420 (1.3) | 30 (2.6) | 1.60 (1.12-2.29) | 8,832 (1.2) | 11 (2.3) | 0.72 (0.13-4.09) |
| Accidents | 312,971 (21.4) | 263 (25.2) | 1.31 (1.16-1.48) | 130,919 (20.7) | 122 (29.1) | 1.24 (0.88-1.74) |

N, number; IR, crude incidence rate per 1000 person-years; HR, hazard ratio; CI, confidence interval.

^a^ Analyses were stratified on full-sister sets.

^b^ HRs were controlled for attained age as the underlying timescale and additionally adjusted for calendar year of birth (1960-1965, 1966-1970, 1971-1975, 1976-1980, 1981-1985, or 1986-1990), educational level (primary school, high school, college and beyond, or unknown), region of residence (south, middle, or north of Sweden), history of injury (yes or no) and psychiatric comorbidities (yes or no).

^c^ The follow-up period of PMD group started from the first clinical diagnosis or indication.

^d^ The follow-up period of PMD group started from the second clinical diagnosis.

**Supplementary Table S9 Associations of premenstrual disorders (PMD) with subsequent risk of injury by excluding person-time preceding the diagnosis among PMD cases.**

|  | **Population analysis** | | | | **Sibling analysis ^a^** | | | |
| --- | --- | --- | --- | --- | --- | --- | --- | --- |
|  | **Reference** | **PMD** | **Model 1** | **Model 2** | **Reference** | **PMD** | **Model 1** | **Model 2** |
|  | **N (IR)** | **N (IR)** | **HR (95% CI) ^b^** | **HR (95% CI) ^c^** | **N (IR)** | **N (IR)** | **HR (95% CI) ^b^** | **HR (95% CI) ^c^** |
| **Any injury** | 333,585 (23.2) | 2,390 (25.6) | 1.37 (1.32-1.43) | 1.31 (1.25-1.36) | 141,677 (22.4) | 973 (25.5) | 1.32 (1.16-1.49) | 1.29 (1.14-1.45) |
| Suicidal behavior | 22,083 (1.3) | 216 (2.1) | 2.27 (1.99-2.60) | 1.66 (1.45-1.90) | 8,911 (1.2) | 89 (2.1) | 1.85 (1.20-2.87) | 1.47 (0.88-2.47) |
| Accidents | 310,271 (21.3) | 2,191 (23.2) | 1.32 (1.27-1.38) | 1.29 (1.23-1.34) | 132,005 (20.7) | 895 (23.2) | 1.29 (1.14-1.47) | 1.28 (1.13-1.45) |

N, number; IR, crude incidence rate per 1000 person-years; HR, hazard ratio; CI, confidence interval.

^a^ Analyses were stratified on full-sister sets.

^b^ HRs were controlled for attained age as the underlying timescale, calendar year of birth (1960-1965, 1966-1970, 1971-1975, 1976-1980, 1981-1985, or 1986-1990), educational level (primary school, high school, college and beyond, or unknown), region of residence (south, middle, or north of Sweden) and history of injury (yes or no).

^c^ HRs were additionally adjusted for psychiatric comorbidities (yes or no).

**Supplementary Table S10 Associations of premenstrual disorders (PMD) with subsequent risk of injury by restricting to untreated specialist-diagnosed PMD.**

|  | **Population analysis** | | | **Sibling analysis a** | | |
| --- | --- | --- | --- | --- | --- | --- |
|  | **Reference** | **PMD** | **HR (95% CI) ^b^** | **Reference** | **PMD** | **HR (95% CI) ^b^** |
|  | **N (IR)** | **N (IR)** |  | **N(IR)** | **N(IR)** |  |
| **Any injury** | 334,524 (23.3) | 1,273 (31.3) | 1.48 (1.40-1.57) | 140,815 (22.4) | 492 (31.1) | 1.34 (1.12-1.59) |
| Suicidal behavior | 22,232 (1.3) | 134 (2.9) | 1.86 (1.57-2.21) | 8,853 (1.2) | 54 (3.0) | 2.33 (1.08-4.99) |
| Accidents | 311,080 (21.4) | 1,169 (28.3) | 1.47 (1.38-1.55) | 131,208 (20.7) | 448 (27.8) | 1.26 (1.05-1.50) |

HR, hazard ratio; CI, confidence interval.

^a^ Analyses were stratified on full-sister sets.

^b^ HRs were controlled for attained age as the underlying timescale, calendar year of birth (1960-1965, 1966-1970, 1971-1975, 1976-1980, 1981-1985, or 1986-1990), educational level (primary school, high school, college and beyond, or unknown), region of residence (south, middle, or north of Sweden), history of injury (yes or no) and psychiatric comorbidities (yes or no).

**Supplementary Table S11 Associations of premenstrual disorders (PMD) with subsequent risk of injury in the population analysis: a simulation analysis by randomly assigning a proportion of PMD diagnoses as false positive.**

|  | **HR (95% CI) ^a^** | | | | | | | |
| --- | --- | --- | --- | --- | --- | --- | --- | --- |
| By percent of false positiveness in PMD diagnosis | | | | | | | | |
|  | 10% | 20% | 30% | 40% | 50% | 60% | 70% | 80% |
| **Any injury** | 1.32 (1.26-1.37) | 1.30 (1.25-1.36) | 1.30 (1.24-1.37) | 1.30 (1.23-1.37) | 1.36 (1.29-1.44) | 1.33 (1.25-1.41) | 1.28 (1.18-1.37) | 1.19 (1.07-1.32) |
| Suicidal behavior | 1.68 (1.46-1.93) | 1.70 (1.46-1.97) | 1.61 (1.37-1.90) | 1.62 (1.36-1.94) | 1.82 (1.52-2.18) | 1.53 (1.23-1.90) | 1.65 (1.30-2.11) | 1.17 (0.79-1.75) |
| Accidents | 1.29 (1.23-1.35) | 1.28 (1.22-1.34) | 1.28 (1.22-1.35) | 1.29 (1.22-1.36) | 1.34 (1.26-1.42) | 1.31 (1.23-1.40) | 1.24 (1.14-1.34) | 1.18 (1.06-1.32) |

HR, hazard ratio; CI, confidence interval.

^a^ HRs were controlled for attained age as the underlying timescale, calendar year of birth (1960-1965, 1966-1970, 1971-1975, 1976-1980, 1981-1985, or 1986-1990), educational level (primary school, high school, college and beyond, or unknown), region of residence (south, middle, or north of Sweden), history of injury (yes or no) and *psychiatric comorbidities (yes or no)*.

**Supplementary Table S12 Associations of premenstrual disorders (PMD) with subsequent risk of injury, stratified by year of birth, educational level, region of residency, and history of injury.**

|  | **Population analysis** | | | | **Sibling analysis ^a^** | | | |
| --- | --- | --- | --- | --- | --- | --- | --- | --- |
|  | **Reference**  **N (IR)** | **PMD**  **N (IR)** | **HR (95% CI) ^b^** | ***P* for interaction** | **Reference**  **N (IR)** | **PMD**  **N (IR)** | **HR (95% CI) ^b^** | ***P* for interaction** |
| **Year of birth** | | | | | | | | |
| 1960-1964 | 50,913 (20.5) | 644 (25.2) | 1.34 (1.23-1.45) | 0.680 | 17,175 (20.4) | 227 (26.3) | 1.27 (0.94-1.60) | 0.459 |
| 1965-1969 | 52,901 (20.0) | 721 (26.7) | 1.34 (1.24-1.44) |  | 23,240 (19.8) | 303 (26.6) | 1.29 (1.02-1.56) |  |
| 1970-1974 | 48,464 (19.2) | 459 (23.3) | 1.27 (1.15-1.39) |  | 22,073 (18.7) | 194 (22.0) | 1.48 (1.08-1.88) |  |
| 1975-1979 | 45,155 (20.1) | 260 (24.3) | 1.29 (1.13-1.45) |  | 20,892 (19.4) | 126 (26.1) | 1.38 (0.90-1.86) |  |
| 1980-1984 | 54,496 (25.2) | 173 (25.8) | 1.25 (1.06-1.44) |  | 25,970 (24.3) | 83 (26.9) | 1.02 (0.58-1.46) |  |
| 1985-1990 | 84,703 (35.4) | 133 (35.5) | 1.18 (0.97-1.39) |  | 32,327 (33.5) | 40 (29.2) | 0.81 (0.32-1.30) |  |
| **Educational level** | | | | | | | | |
| Primary | 67,689 (16.9) | 684 (22.5) | 1.43 (1.32-1.54) | 0.041 | 29,429 (16.6) | 290 (23.1) | 1.57 (1.21-1.92) | 0.117 |
| High School | 130,902 (21.1) | 1,224 (25.0) | 1.25 (1.18-1.32) |  | 56,227 (20.7) | 513 (25.7) | 1.24 (1.03-1.44) |  |
| College and beyond | 64,168 (29.0) | 356 (33.1) | 1.30 (1.16-1.44) |  | 28,932 (28.0) | 134 (29.9) | 1.11 (0.75-1.46) |  |
| Unknown | 73,873 (36.5) | 126 (38.6) | 1.20 (0.98-1.42) |  | 27,089 (34.6) | 36 (31.6) | 0.82 (0.32-1.33) |  |
| **Region of residency** | | | | | | | | |
| South | 87,194 (26.5) | 537 (29.2) | 1.30 (1.19-1.42) | 0.945 | 37,537 (25.3) | 227 (29.5) | 1.18 (0.87-1.48) | 0.392 |
| Middle | 184,913 (22.8) | 1,400 (24.9) | 1.30 (1.23-1.37) |  | 76,485 (21.9) | 564 (24.9) | 1.38 (1.16-1.61) |  |
| North | 64,525 (21.2) | 453 (24.0) | 1.31 (1.19-1.44) |  | 27,655 (20.5) | 182 (23.3) | 1.14 (0.84-1.45) |  |
| **History of injury** | | | | | | | | |
| No | 284,496 (21.5) | 1,556 (20.8) | 1.25 (1.18-1.31) | 0.003 | 120,796 (20.9) | 645 (21.1) | 1.23 (1.06-1.40) | 0.257 |
| Yes | 52,136 (42.9) | 834 (45.0) | 1.42 (1.32-1.52) |  | 20,881 (40.1) | 328 (43.5) | 1.45 (1.10-1.81) |  |

N, number; IR, crude incidence rate per 1000 person-years; HR, hazard ratio; CI, confidence interval.

^a^ Analyses were stratified on full-sister sets.

^b^ HRs were controlled for attained age as the underlying timescale and additionally adjusted for calendar year of birth (1960-1965, 1966-1970, 1971-1975, 1976-1980, 1981-1985, or 1986-1990), educational level (primary school, high school, college and beyond, or unknown), region of residence (south, middle, or north of Sweden), history of injury (yes or no) and psychiatric comorbidities (yes or no).
